# Evaluation of the Probiotic Potential of *Lactobacillus acidophilus* Strain JB2CON

**DOI:** 10.1155/bmri/4454851

**Published:** 2026-04-21

**Authors:** Sk. Md. Jakaria Al-Mujahidy, Md. Ramjan Sheikh, Md. Ekhlas Uddin, Shahidur Rahman, A. N. M. Mamun-Or-Rashid, Md. Liton Miah

**Affiliations:** ^1^ Environmental Health and Synthetic Biology Research Lab (EHSBR), Wazed Miah Science Research Centre (WMSRC), Jahangirnagar University, Dhaka, Bangladesh, juniv.edu; ^2^ Department of Biochemistry and Molecular Biology, Hajee Mohammad Danesh Science and Technology University, Dinajpur, Bangladesh, hstu.ac.bd; ^3^ Department of Biochemistry and Molecular Biology, Gono Bishwabidyalay, Dhaka, Bangladesh; ^4^ Dairy Development Research Project, Bangladesh Livestock Research Institute Regional Station, Sirajganj, Bangladesh; ^5^ University of Pittsburgh, Pittsburgh, Pennsylvania, USA, pitt.edu

**Keywords:** 16S gene sequencing, antimicrobial properties, *E. coli*, *L. acidophilus* JB2CON, probiotic, shelf life

## Abstract

**Background:**

Probiotics have attracted much attention recently because of their potential health benefits and aim at correcting the imbalance of gut microbiota. Dairy fermented foods play an important role as a probiotic carrier, and fermented dairy products like dahi are well known for their ability to maintain the viability of bacteria and their acceptance by consumers. Nevertheless, the production of low‐cost, locally manufactured probiotic products that possess antimicrobial activity against food pathogens is relatively scarce. The principal goal of this current study was to isolate a new potential probiotic strain to prepare contamination‐free probiotic dahi in a dosage form with a desired shelf life.

**Method:**

To achieve our target, firstly, probiotic dahi was prepared by a probiotic strain, *Lactobacillus acidophilus* JB2CON (OM909067) (identified by 16S sequencing) isolated from natural dahi in Dhaka, Bangladesh. Probiotic potential was assessed by transmission electron microscopy, acid and bile tolerance, hemolytic activity, antibiotic sensitivity, and antagonistic activity against pathogens, while sensory acceptance of the probiotic dahi was evaluated by 16 participants using a 9‐point hedonic scale.

**Results:**

The isolate exhibited five probiotic features by in vitro test and had an antagonistic effect against four different bacterial pathogens and *Candida albicans*. More interestingly, it inhibited *Escherichia coli* (*d* *i* *a* *m* *e* *t* *e* *r* *of* *z* *o* *n* *e* *of* *i* *n* *h* *i* *b* *i* *t* *i* *o* *n* = 15.75 ± 0.35) the most. In the time‐kill analysis method, *E. coli* was killed within 5.50 h of fermentation. In the antibiotic sensitivity test, the isolated bacterial species exhibited clear inhibition, with ampicillin producing the largest zone (69.33 ± 11.06 mm), followed by tylosin (67.33 ± 6.43 mm). In the acid tolerance test, the probiotic count decreased by ~3.3 log units after 24 h, while bile salt exposure (0.025% and 0.5% oxgall) reduced viable counts in a concentration‐dependent manner. The probiotic dahi prepared in this study was rated as “moderately liked” by 16 participants using a 9‐point hedonic scale. Stability testing identified 14 days as the optimal shelf life, with increased probiotic counts and no adverse effect of minor pH changes on dahi taste.

**Conclusion:**

The most significant impact of this research is that the probiotic dahi of our experiment might be used as a cheap option for preventing pathogenic *E. coli*‐related foodborne diseases worldwide, especially in underdeveloped countries like Bangladesh.

## 1. Background

Probiotics benefit human health, and *Lactobacillus acidophilus* is the most popularly consumed probiotic [1]. Research has demonstrated that *L. acidophilus* can produce potential probiotic effects in humans, such as acting as a barrier against pathogens, recovering from lactose intolerance, boosting immune response, and decreasing cholesterol levels [2]. *L. acidophilus* can colonize the intestines of humans and inhibit pathogens, such as *Escherichia coli*, *Salmonella typhimurium*, and *Streptococcus aureus*. These pathogens also inhabit the intestines [1, 3]. These probiotics also prevent diarrheal infections [4]. According to the last global burden of disease study, approximately 2.39 billion people were infected by diarrheal cases globally, and nearly 0.53 million under‐five children died yearly. In lower‐ and middle‐income (LMI) countries, specifically Bangladesh, incidence and case‐fatality ratios are much higher than those of developing countries [5]. In Bangladesh, the average cost per episode was $67.18, while the moderate inpatient and outpatient charges were $110.51 and $23.62, respectively. The price was substantially the largest for impoverished households, 21.45% of household income, compared to 4.21% of the prosperous people [5]. *L. acidophilus* can be a cost‐saving option for preventing diarrheal disease and pathogenic *E. coli*‐related other food‐borne diseases in billions of people worldwide.

Potential probiotic strains are vital for killing pathogens in the intestines. In Bangladesh, most of the marketed probiotics are purchased from foreign countries. There is very little research on isolated probiotics in Bangladesh [6]. Moreover, assessment of the potential probiotic traits of a strain is essential for claiming it as a probiotic strain [5, 7]. Isolation of potential probiotics from Bangladeshi natural sources is significant in boosting the immunity of the masses of this geographic territory. This study focused on isolating a new probiotic strain of *L. acidophilus* from Bangladeshi natural sources.

Probiotic dahi is a good option for delivering probiotics to the intestines. Dahi is a customary yogurt or fermented milk product in the Indian subcontinent, usually produced from cow′s milk, buffalo, or goat′s milk. It is consumed around the Indian subcontinent [8]. We can get two benefits from probiotic dahi: one is a test, and the other is the probiotic culture. In the Bangladeshi market, no probiotic dahi has specific probiotic bacteria levels [5]. The shelf life of dahi is another vital factor for maintaining its quality up to the expiration date. There is a correlation between pH, temperature, probiotic growth, and contamination in dahi [9, 10]. The preparation of dahi in unhygienic environments and contaminated starter cultures can spoil dahi and cause food poisoning. Shelf life is dependent on this issue. Shelf life depends on another factor: the total live probiotic count [11, 12].

The main target of this topical investigation was to prepare a probiotic dahi having potential probiotic *L. acidophilus* in a dosage form; that is, the number of probiotics would not be less than a certain number of colony‐forming units (CFU) per gram of dahi. Secondly, we aimed to find the capability of dahi to inhibit the growth of contaminants. Information on probiotic growth and inhibition of microbial contamination would be used to determine the shelf life of probiotic dahi. A preliminary version of this study has been previously deposited as a preprint on http://Preprints.org [13].

## 2. Methods

### 2.1. Probiotic Isolation and Identification

#### 2.1.1. Probiotic Isolation

Three independent dahi samples (biological replicates, *n* = 3) were used for probiotic isolation from Dhaka, Bangladesh, following Vinderola et al. [14]. One milliliter of a 10^−9^ decimal diluted sample (suspended in standard 0.9% [*w*/*v*] saline solution) was spread on 20–25 mL of MRS (de Man–Rogosa–Sharpe, Oxoid, United Kingdom) agar medium, and the plates were incubated for 24–72 h at 37°C. The most distinguished common bacterial colonies were selected from MRS agar media based on morphology. To purify colonies, the isolates were streaked on the same media, and finally, the pure colonies were transferred to MRS broth with 15% glycerol for further research. The Leica MZ9.5 (Germany), a potent stereo microscopic instrument with a fantastic 9.5:1 zoom ratio and magnification capabilities as high as 480x, was used to analyze the colony morphology.

#### 2.1.2. Gram Staining Test, 16S Gene Sequencing, and Phylogenetic Tree

The selected colonies were examined using the Gram staining protocol using the Coica [15] method and then observed under a light microscope at 100x resolution [15]. According to the manufacturer′s protocol, the MRS agar cultured colonies were used for genomic DNA isolation with the phenol‐chloroform chemical lysis method. After DNA extraction, the concentration and purity of DNA were checked using a NanoDrop spectrophotometer ND2000 (Thermo Scientific, United States). The 260/280 ratio (absorbance at 260 and 280 nm) indicated the purity of the DNA. The A260/A280 ratio of all DNA samples was > 1.8, indicating acceptable purity. In this study, 16S rDNA amplification and sequencing were performed based on the methodology described previously by Rahman et al. [16]. The universal 16S rRNA primer set for polymerase chain reaction (PCR) amplification was as follows: 27f (5 ^′^‐AGAGTTTGATCCTGGCTCAG‐3 ^′^ and 1492r (5 ^′^‐GGTTACCTTGTTACGACTT‐3 ^′^) [16]. The neighbor‐joining method inferred the phylogenetic tree [17]. The bootstrap consensus tree inferred from 1000 replicates represents the evolutionary history of the taxa analyzed [18]. This analysis involved 12 nucleotide sequences. Evolutionary analyses were conducted in MEGA11 [19].

#### 2.1.3. Cellular Morphology Analysis Under Transmission Electron Microscope

The grids were hydrophilized using a plasma cleaner (low power, 12 s) prior to sample application, followed by [20]. After that, a tabletop centrifuge was put to work to spin the bacterial culture for 2 min at 2000–4000 rpm. Ten microliters of pure water was used for the resuspension of the particle. After applying the specimen to the grid, it was left in place for 1 min. The samples were incubated for 1 min, stained with a 1.8% uranyl acetate solution, and then air‐dried. The photos of bacterial samples were captured with a JEM 1010 electron transmission microscope (JEOL). TEM analysis was performed on two independently prepared samples.

### 2.2. Probiotic Potentiality and Safety Test

#### 2.2.1. Antibiotic Sensitivity Test by Agar Well Diffusion Method

An antibiotic sensitivity test was conducted against the probiotic isolated JB2CON, following the procedure of Sharma et al. [21] with some modifications. Eight milliliters of MRS agar (with log 8 CFU/mL of the isolated JB2CON) was overlaid on previously solidified MRS agar [21]. Cefuroxime (30 *μ*g/mL), gentamycin (10 *μ*g/mL), chloramphenicol (30 *μ*g/mL), erythromycin (15 *μ*g/mL), clindamycin (10 *μ*g/mL), tylosin (30 *μ*g/mL), ampicillin (10 *μ*g/mL), vancomycin (30 *μ*g/mL), kanamycin (30 *μ*g/mL), streptomycin (10 *μ*g/mL), and tetracycine (30 *μ*g/mL) were filled in a 7 mm diameter well in MRS agar media with an upper layer of the probiotic bacterial strain. The antibiotics selected were due to their clinical significance, food safety concerns, and the common resistance profiles reported in probiotic bacteria. The test Petri dishes were incubated for 24 h at 37°C. Then, the diameters of zones were interpreted following the Clinical and Laboratory Standards Institute (CLSI) guidelines. Negative control (without bacteria) was prepared by plating on sterile MRS agar and without antibiotics. All antimicrobial susceptibility tests were conducted in three independent replicates. The mean and standard deviation (SD) of the zone of inhibition (ZOI) were analyzed by R Studio (V. 4.2.3).

#### 2.2.2. Hemolysis Test

Blood agar (BD, United States) plates were streaked with MRS broth containing JB2CON strain cultures to evaluate hemolytic activity and incubated for 72 h at 37°C. Next, each of the plates was inspected to see if the *Lactobacillus* colonies were surrounded by any greenish (*α*‐hemolysis) or clean (*β*‐hemolysis) hemolytic zones or if there were none at all (*γ*‐hemolysis). The hemolysis assay was repeated three times (*n* = 3), and the positive reaction was recorded as a qualitative test [22]. Two replicates were used for statistical analysis.

#### 2.2.3. Acid Tolerance

The acid tolerance of the isolate JB2CON was tested following the method of Ortakci et al. [23] with some modifications. About 0.2 g NaCl plus 0.7 mL HCl (stock solution) and sufficient water were used to make 100 mL simulated gastric juice (SGJ of pH 1.4). SGJ and sterilized dahi were mixed in a ratio of 1:4 to make a solution of pH 2.35 (pH < 3) [23]. This final solution was used for analyzing the capability of the isolate JB2CON in our study to tolerate acidity (pH < 3). One milliliter of probiotic isolate (1.28 × 10^8^ CFU/mL) was added to the 9 mL final solution of SGJ and dahi. Viable counts were determined after 24 h of incubation at 37°C. Cells incubated in MRS broth without SGJ served as the control. The experiment was repeated twice. The mean and SD of CFU were analyzed by R Studio (V. 4.2.3).

#### 2.2.4. Bile Salt Tolerance

With minor adjustments, the bile tolerance of the isolated JB2CON was assessed using the methodology of Hassanzadazar et al. [24]. The isolate JB2CON was incubated for 24 h at 35°C in MRS broth. Following this time, the cells were suspended using a slow vortex. After that, 1% cell suspension broth was mixed with the MRS broth containing bile salt (0.5% and 0.25% oxgall). The isolate JB2CON′s viability was assessed after 8 h of incubation by culturing it in Petri dishes using the spread plate technique, which was then incubated for 5 days at 35°C. MRS broth without bile salt served as the negative control. Each experiment was conducted in duplicate. The mean and SD were analyzed by R Studio (V. 4.2.3).

#### 2.2.5. Antimicrobial Activity Test by Agar Well Diffusion Method

The antimicrobial activity test of the isolate JB2CON was conducted following the process of Begum et al. [6] with some modifications. Antagonistic effects against six pathogenic microorganisms were tested in TSA media [6]. About 100 *μ*L of supernatant of the cell suspension broth of the isolated JB2CON (final pH 3.98 after 48 h incubation at 35°C) was placed in a 7 mm diameter well in TSA media with a lawn of pathogenic microorganisms. Two replicates of antagonistic tests were conducted against each pathogen. *S. typhimurium* ATCC 14028, *S. aureus* ATCC 6538, *E. coli* ATCC 8739, *Bacillus subtilis* ATCC 6633, *Pseudomonas aeruginosa* ATCC 142, and *Candida albicans* ATCC 10231 were bought from a local supplier in Dhaka, Bangladesh. TSA plates without supernatant served as the negative control. All assays were performed in duplicate. The mean and SD of the ZOI of antimicrobial activity were analyzed by R Studio (V. 4.2.3).

#### 2.2.6. Antimicrobial Activity Test by Time‐Kill Assay Against Unknown Contamination

In the agar diffusion assay, we used specific pathogens of ATCC cultures, although in the time‐kill assay, we employed naturally occurring environmental contaminants. The antimicrobial activity test was performed with some modifications by the time‐kill assay described by Prabhurajeshwar and Kelmani [25]. For this assay, random contaminations and all ingredients for dahi (pasteurized milk, sugar, plus the isolate JB2CON) were added to each container before starting incubation. The contaminations were added from natural sources (water, glassware, sucrose, pasteurized milk, etc.) to each container. Three containers were prepared for three different temperatures: ambient room temperature, 35°C, and 44°C. After 21 h of incubation, microbial contaminants were observed for the dahi samples in a 100‐fold dilution. Plates showing more than 300 CFU were considered too numerous to count (TNTC). Containers prepared without the probiotic isolate JB2CON served as contamination controls. All time‐kill experiments were conducted in duplicate.

For the contamination test, we prepared media and conducted growth promotion tests according to the guidelines of the nutrient and dietary supplements section in the United States Pharmacopeia (USP) [26]. Total nonlactic contamination (TNLC) was tested in Tryptic Soy Broth and Tryptic Soy Agar, and Total Yeast and Mold Count (TYMC) was tested in Tryptic Soy Broth and Sabouraud Chloramphenicol Agar. Moreover, specific pathogens, particularly *Salmonella*, were tested on selective media, such as Rappaport Vassiliadis Salmonella Enrichment Broth and Xylose Lysine Deoxycholate Agar (XLD agar). The presence of *E. coli* was observed in MacConkey Broth and MacConkey Agar. Furthermore, the presence of *S. aureus* and *P. aeruginosa* was checked on Mannitol Salt Agar and Pseudomonas Cetrimide Agar, respectively [27].

#### 2.2.7. Analysis of Milk for Preparation of Dahi

A cow′s milk sample was collected from Dhaka Milk Industry, Dhaka, Bangladesh. Milk was analyzed for four properties, such as lactose, protein, fat, and minerals. Although these four properties were tested in the Dhaka Milk Industry and the result was presented on their label, we retested these properties once by a milk analyzer (LactoScan LW, Milkotronic Ltd.) following the method of AOAC (AOAC 13th edition, 2012), followed by [28]. All measurements were performed once for verification purposes.

#### 2.2.8. Preparation of Probiotic Dahi

Then, 0.5 L of cow milk containing 30.0 g of sucrose was concentrated to 0.4 L through the application of heat. Subsequently, the temperature of the milk was reduced to approximately 45°C. Approximately 1.0 g of JB2CON culture (containing ~10^8^ CFU/g) was added to each container. The containers were maintained at 44°C until the dahi reached a solid state. The pH of dahi was measured following the solidification process.

### 2.3. Shelf Life Study at Storage Conditions

For selecting the suitability of the shelf life of probiotic dahi, antimicrobial activity tests and shelf life tests were carried out simultaneously. An antimicrobial activity test was performed with some modifications by the time‐kill assay described by Begum et al. [6]. For this, four containers (each one containing probiotic dahi) were incubated at 44°C for 5.50 h and then stored at freezing temperature (2°C–8°C) for 14 days. Container‐1 was filled with all ingredients of dahi (pasteurized milk, sugar, plus the isolate JB2CON) aseptically, and other containers contained the components of Container‐1 plus additional contaminations. Container‐2 was allowed with unknown contamination (natural) and probiotic isolate, Container‐3 was filled with *C. albicans* ATCC 10231 plus probiotic isolate, and Container‐4 was inoculated with *E. coli* ATCC 8739 plus probiotic isolate. The shelf life of probiotic dahi was tested for 14 days in refrigerated (2°C–8°C) conditions. *L. acidophilus* should be present at levels of at least 10^5^–10^6^ CFU/mL at the time of consumption to confer probiotic effects [2]. As a result, we decided that the dosage of our probiotic dahi for the duration of its shelf life should be at least 10^6^ CFU/mL. The total probiotic count after 14 days was compared with that on the first day at freezing temperature. This comparison was conducted for TNLC, TYMC, *C. albicans* ATCC 10231, and *E. coli* ATCC 8739. The tests were performed twice, as identical counts were observed in both replicates, and SD was not calculated.

### 2.4. Consumer Acceptance of Dahi

Probiotic dahi was prepared according to the process described in Section 2.2.8 and incubated at 44°C for 5.50 h. Consumer acceptance was assessed by a sensory test conducted by the method of Hashim et al. [29]. A 9‐point hedonic scale was used, such as *like extremely* = 9, *like very much* = 8, *like moderately* = 7, *like slightly* = 6, *neither like nor dislike* = 5, *dislike slightly* = 4, *dislike moderately* = 3, *dislike very much* = 2, and *dislike extremely* = 1 [29]. A total of 16 untrained panelists (*n* = 16) participated in four groups, with four people in each group. All participants provided verbal consent prior to sensory evaluation.

## 3. Results

### 3.1. Probiotic Isolation and Identification

#### 3.1.1. Probiotic Isolation

**Figure 1:**
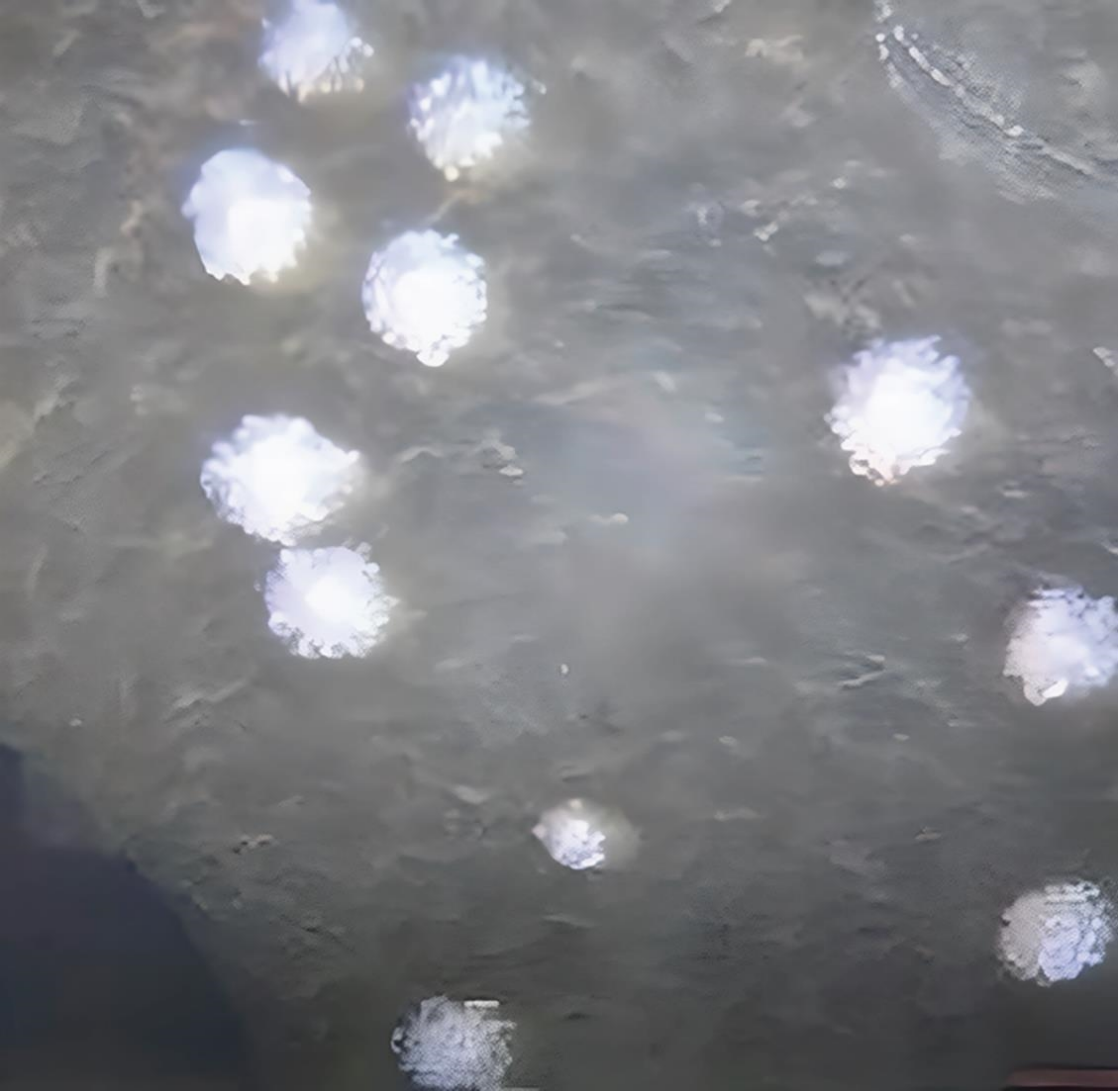
The colony morphology of the colony JB2CON was observed under a stereo microscope, Leica MZ9.5 (Germany).

In MRS agar media, several colonies showed *Lactobacillus* characteristics from morphological viewpoints (Figure 1). This study found colonies were white, round, raised, and translucent.

#### 3.1.2. Gram Staining Test, 16S Gene Sequencing, and Phylogenetic Tree

**Figure 2:**
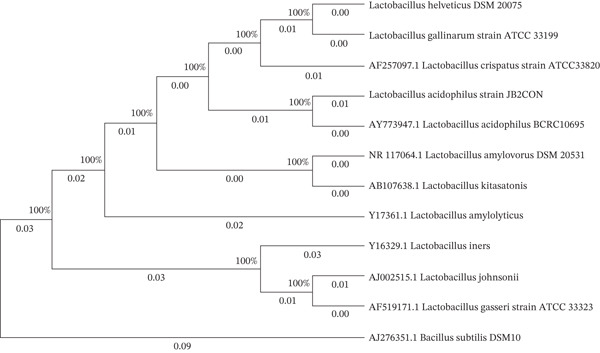
The phylogenetic tree was inferred using the neighbor‐joining method. The bootstrap consensus tree inferred from 1000 replicates represents the evolutionary history of the taxa analyzed. Evolutionary analyses were conducted in MEGA11. In the tree with the *L. acidophilus* subgroup, our isolated strain JB2CON was placed with AY773947.1. *L. acidophilus* BCRC1069.

Gram staining showed the cells were Gram‐positive and long rods. According to the 16S gene sequence, the colony JB2CON was identified as *L. acidophilus* (Accession No. OM909067 and strain JB2CON). In the *L. acidophilus* subgroup tree, our isolated strain JB2CON was placed with AY773947.1 L*. acidophilus* BCRC1069. Therefore, the strain JB2CON was identified as *L. acidophilus.* The tree was rooted with the 16S rRNA gene from AJ276351.1 *B. subtilis* DSM10 (Figure 2).

#### 3.1.3. Cellular Morphology Analysis Under Transmission Electron Microscope

**Figure 3:**
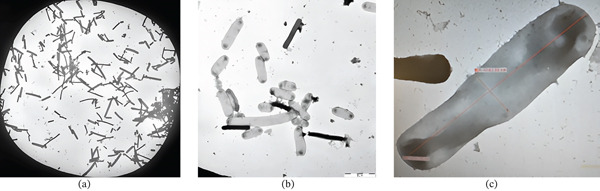
The cellular morphology of *L. acidophilus* strain JB2CON in MRS broth was observed under a JEM 1010 electron transmission microscope (JEOL). The left image (1000‐fold magnification) depicted rod‐shaped free cells and cells in chains. The middle image demonstrated rod‐shaped cells of varying lengths and widths at 5000‐fold magnification. The right picture showed a single cell of the strain JB2CON at 30,000‐fold magnification (length: 4.76 *μ*m and width: 1.14 *μ*m).

Under a transmission electron microscope, microorganisms with a rod shape and sizes between 2 and 5 *μ*m were seen. In the course of our research, we identified rounded‐end rods that had been found in pairs, short chains, or even as single cells (Figure 3).

### 3.2. Probiotic Potentiality and Safety Test

In the current study, we tested three probiotic features of the isolate, *L. acidophilus* JB2CON (Accession No. OM909067).

#### 3.2.1. Antibiotic Sensitivity Test

In this study, *L. acidophilus* JB2CON (OM909067) demonstrated a ZOI exceeding 20 mm in diameter. According to the description of the CLSI, they were susceptible to cefuroxime (100 *μ*L/30 *μ*g), gentamycin (100 *μ*g/mL), chloramphenicol (100 *μ*g/mL), erythromycin (15 *μ*g/mL), clindamycin (10 *μ*g/mL), tylosin (30 *μ*g/mL), ampicillin (10 *μ*g/mL), vancomycin (30 *μ*g/mL), kanamycin (30 *μ*g/mL), streptomycin (10 *μ*g/mL), and tetracycine (30 *μ*g/mL) (Table 1). Every test for antibiotic sensitivity was performed thrice.

**Table 1:** Antibiotic sensitivity test.

Antibiotic	Dose	ZOI
Cefuroxime	30 *μ*g/mL	33.0 ± 4.24
Gentamycin	10 *μ*g/mL	22.0 ± 2.0
Chloramphenicol	30 *μ*g/mL	48.67 ± 2.31
Erythromycin	15 *μ*g/mL	31.0 ± 1.0
Clindamycin	10 *μ*g/mL	30.0 ± 6.0
Tylosin	30 *μ*g/mL	67.33 ± 6.43
Ampicillin	10 *μ*g/mL	69.33 ± 11.06
Vancomycin	30 *μ*g/mL	48.67 ± 4.16
Kanamycin	30 *μ*g/mL	21.33 ± 2.31
Streptomycin	10 *μ*g/mL	20.67 ± 1.15
Tetracycine	30 *μ*g/mL	42.0 ± 2.0

#### 3.2.2. Hemolysis Test

Hemolytic activity was not observed for the strain JB2CON. The isolate was identified as *γ*‐hemolytic or nonhemolytic because no distinct transparency or greenish zone encircled its colonies on the blood agar Petri dishes.

#### 3.2.3. Acid Tolerance

The acid tolerance test was conducted in a solution of pH 2.35 because the pH of the human stomach in its natural state is between 1.5 and 3.5 [30]. The probiotic count in the tested solution decreased to (5.98 ± 0) × 10^3^ CFU/mL after 24 h from (1.28 ± 0) × 10^7^ CFU/mL of the initial count. Therefore, the probiotic *L. acidophilus* JB2CON (OM909067) was moderately tolerant to the pH of gastric juice.

#### 3.2.4. Bile Salt Tolerance

The good bacteria in the human colon, known as probiotics, must adjust to bile salts. Oxgall was used to assess the bile salt tolerance of the isolate. After incubating the isolate for 8 h, we cultivated it on plates and saw growth on Petri dishes. When 0.025% and 0.5% oxgall were added, the CFU per milliliter decreased from (1.37 ± 1.52) × 10^6^ to (1.53 ± 2.14) × 10^5^ and (1.40 ± 2.25) × 10^4^, respectively, while it increased to (2.42 ± 3.11) × 10^6^ in the control broth. For a bile sensitivity test in MRS broth, Ortakci et al. [23] selected some microbial cultures with bile concentrations as high as 0.3% as the test and 0% as the control. The present study contained a higher concentration of viable bacteria compared to that reported by Ortakci et al. [23]. Thus, the isolated JB2CON can be considered a bile salt–resistant strain under in vitro conditions, and it may survive gastrointestinal stress of bile.

#### 3.2.5. Antagonistic Test

After 48 h of incubation, the ZOI was measured. The inhibition zones of more than 20 mm, 10–20 mm, and less than 10 mm were accepted as decisive, intermediate, and low inhibition, respectively. In our study, *L. acidophilus* JB2CON (OM909067) had an intermediate inhibition against the tested pathogens (Table 2). We found the widest ZOI (14.5 mm) against *E. coli* ATCC 8739 and no ZOI against *C. albicans* ATCC 10231 by the agar well diffusion method. That is why we conducted further anti‐*E. coli* and anti‐*C. albicans* experiments during the time‐kill assay method during the shelf life test.

**Table 2:** Antagonistic test.

Bacteria	Diameter of ZOI (mm)
*S. typhimurium* ATCC 14028	13.5 ± 0.5
*S. aureus* ATCC6538	12.0 ± 0.0
*E. coli* ATCC 8739	15.75 ± 0.35
*B. subtilis* ATCC 6633	12.25 ± 0.35
*P. aeruginosa* ATCC 1427	12.0 ± 1.41
*C. albicans* ATCC 10231	0.0

#### 3.2.6. Antimicrobial Activity Test by Time‐Kill Assay for Unknown Contamination

TNLCs were TNTC on TSA media. On the other hand, no CFU of TYMC was observed on SCA media at 35°C and 44°C, whereas TNTC was observed at ambient room temperature on this media (Table 3). Further dilution was not performed for counting the exact CFU of unknown contamination because the tested samples did not comply with the USP. According to USP nutritional and dietary supplement guidelines, the microbial limit for TAMC is 1000 CFU/mL, and for TYMC, it is 100 CFU/mL. On the contrary, identical colonies of *E. coli*, *S. aureus*, *Salmonella* spp., and *P. aeruginosa* were not found on selective media (Table 4). The samples complied with USP specifications in terms of specific pathogenic microorganisms. These results indicate possible preservative effects of probiotic dahi produced using *L. acidophilus* JB2CON (OM909067) against some pathogenic bacteria under the prevailing experimental conditions, albeit there are some resistant unknown bacteria, and fungi can grow in dahi naturally. Thus, precautions should be taken to prepare dahi and select a starter culture.

**Table 3:** Effect of pH (for specific incubation temperature) on total microbial contaminants.

pH	Temperature	CFU/mL in TSA	Types of morphology in TSA	CFU/mL in SCA	Types of morphology in SCA
4.74	Ambient room temperature	TNTC	3	TNTC	1
3.95	35°C	TNTC	2	< 100	0
3.75	44°C	TNTC	2	< 100	0

**Table 4:** Effect of pH (for certain incubation temperatures) on specific pathogenic microbial contaminants.

Microorganism	Media	pH	Temperature	Identical colony
*E. coli*	MacConkey Broth and MacConkey Agar	4.74	Ambient	No growth
		3.95	35°C	No growth
		3.75	44°C	No growth

*S. aureus*	Mannitol Salt Agar	4.74	Ambient	No growth
		3.95	35°C	No growth
		3.75	44°C	No growth

*Salmonella* sp.	Rappaport Vassiliadis Salmonella Enrichment Broth and XLD agar	4.74	Ambient	No growth
		3.95	35°C	No growth
		3.75	44°C	No growth

*P. aeruginosa*	Pseudomonas Cetrimide Agar	4.74	Ambient	No growth
		3.95	35°C	No growth
		3.75	44°C	No growth

#### 3.2.7. Analysis of Milk for the Preparation of Dahi

Milk quality parameters, such as protein and fat contents, influence milk′s titrable acidity and, subsequently, dahi′s titrable acidity and pH [9]. Thus, we tested the milk properties of cow milk. Fat content was not less than 2%, and protein content was 3.5% (Table 5).

**Table 5:** Milk properties.

Serial no.	Ingredients	Amount
1	Fat	2.67%
2	SNF (solids‐not‐fat)	8.6%
3	Protein	3.10%
4	Lactose	4.1%
5	Specific gravity	1.027
6	pH	6.81
7	Conductivity	4.1
8	Added water	2%
9	Temperature	24.5
10	Freezing point	−0.693
11	Salts	0.85%

#### 3.2.8. Preparation of Probiotic Dahi

The solidification was found in the case of probiotic dahi at 44°C, and it happened within 4 h when the pH reached 4.75. *L. acidophilus* is well suited for living in a dairy medium, as fermented milk is the ideal delivery method for introducing *L. acidophilus* into a gut microbiome [31].

### 3.3. Shelf Life Study at Storage Conditions

A total of four containers (containing probiotic dahi) were incubated at 44°C for 5.50 h and then stored in a refrigerator (2°C–8°C) for 14 days (Table 6). The previous study confirmed that the viability of *L. acidophilus* cells stored in a refrigerator (4°C) is higher than that of cells stored at room temperature. Thus, in the current study, we used a fridge to store our probiotics. No *E. coli* cell was found alive on TSA media after 5.50 h, while *C. albicans* cells increased gradually and soared to 114 CFU/g after 14 days. This finding reveals that *L. acidophilus* JB2CON (OM909067) in this storage period at the refrigerated condition had an inhibitory effect on *E. coli*. TNLC and TYMC also increased steadily in TSA and SCA after 14 days. It indicates that probiotic dahi prepared by *L. acidophilus* JB2CON (OM909067) can be a natural preservative for inhibiting the growth of *E. coli*. However, some resistant bacteria and fungi can grow in dahi naturally. Thus, precautions should be taken to prepare dahi and select a starter culture. For setting shelf life, these factors should be considered carefully.

**Table 6:** Time and pH on probiotic bacterial count and contamination count at storage conditions.

Day and pH	Probiotic count (CFU/30 mL)	Contamination on TSA (CFU/mL)	Contamination on SCA (CFU/mL)	*C. albicans* (CFU/mL)	*E. coli* (CFU/mL)
	Container‐1	Container‐2	Container‐2	Container‐3	Container‐4
1 and 3.65 ± 0	60 ± 4.24 × 10^9^	TNTC	4.5 ± 0.71	94.5 ± 0.71	00
3 and 3.65 ± 0	72.0 ± 0 × 10^9^	TNTC	5.0 ± 0	106.0 ± 0	00
14 and 3.59 ± 0	170.0 ± 0 × 10^9^	TNTC	121.0 ± 0	114.0 ± 0	00

The container with pasteurized milk had no microbial contamination. Interestingly, in this container, the probiotic count of dahi increased 2.98‐fold and soared to 170.0 ± 0 × 10^9^ CFU/30 mL after Day 14 from 60 ± 4.24 × 10^9^ CFU/30 mL on Day 1. We decided that our probiotic dahi should contain at least 10^6^ CFU/mL for its shelf life, following Alberts′ [2] suggestions. However, our yogurt with probiotics was far more concentrated for up to 14 days. On the other hand, dahi′s pH increased slightly (0.06) from 3.65 ± 0 to 3.59 ± 0 (Table 6). This indicates that CFU has increased gradually for 14 days, but pH has not increased noticeably. This slight change in pH cannot affect dahi′s taste (sourness/acidity). Therefore, a shelf life of up to 14 days appears suitable for maintaining viable probiotic counts. According to the findings of our study, to keep a shelf life of 14 days, probiotic dahi should be prepared from pasteurized cow milk.

### 3.4. Consumer Acceptance of Dahi

The overall consumer acceptance of dahi was satisfactory (Table 7). A 9‐point hedonic scale for the sensory test of probiotic dahi was applied to 16 people (four groups). The texture of dahi was neither liked nor disliked by the people of the four groups. In the probiotic dahi, we did not add any extra additives, and the fermentation time was reduced to decrease the sourness or acidity of the dahi. Therefore, our prepared probiotic dahi was soft. However, the groups of people liked the taste of the probiotic dahi moderately, and they liked the color, sweet, and sour slightly or moderately.

**Table 7:** Sensory test by a 9‐point hedonic scale.

Group	Favor	Color	Sweet	Sour	Texture
1	7.25 ± 1.50	6.75 ± 0.95	6.0 ± 0.81	6.0 ± 0.81	4.5 ± 0.58
2	7.0 ± 0.81	7.0 ± 0.81	7.0 ± 0.81	7.0 ± 0.81	5.0 ± 0
3	7.5 ± 1.29	7.5 ± 1.29	7.5 ± 1.29	7.5 ± 1.29	5.0 ± 0
4	7.0 ± 0.81	6.0 ± 0.81	6.0 ± 0.81	6.0 ± 0.81	5.0 ± 0

*Note:* A 9‐point hedonic scale was used, where 1 = *dislike extremely* and 9 = *like extremely*; mean ± SD was calculated for four people in each group.

## 4. Discussion

*L. acidophilus* is the most consumed probiotic, providing numerous beneficial health effects [1]. Therefore, this bacterium must have an extended shelf life. Colony morphology, staining, 16S gene sequencing, and antimicrobial sensitivity tests are the crucial steps to analyze the shelf life of *L. acidophilus* [32]. This facultative anaerobic bacterium showed white, round, raised, and translucent colonies in the MRS agar [33, 34]. *L. acidophilus* is Gram‐positive and long rod‐shaped by Gram staining [34]. The phylogenetic tree was constructed with strains of 10 species in the *L. acidophilus* subgroup, and this desired species was found [35]. The genetic material of *L. acidophilus* directly affects its well‐being, especially regarding gut health, immune function, and avoiding gastrointestinal diseases [36, 37]. Its probiotic potential is demonstrated by its antibacterial compound production, lactose fermentation, immune system modulation, and bioactive substance production capabilities [38, 39]. For these reasons, *L. acidophilus* is a valuable strain for medicinal and nutritional purposes, especially for promoting good gut health and the immune system′s effectiveness.

Our investigation revealed rod‐shaped bacteria measuring 2–5 *μ*m, examined with a transmission electron microscope. Some rod‐shaped cells displayed rounded ends, indicating a potential physiological or structural adaptation [40]. The configuration of these cells may signify a particular pattern of growth, potentially influenced by environmental variables or inherent genetic pathways [41]. *L. acidophilus* is the most common probiotic in the world, as demonstrated by [42]. Our investigation found that the identified probiotic species has a ZOI greater than 20 mm in diameter, indicating a high antibiotic sensitivity level. The hypothesis that JB2CON may have a wide range of antibiotic susceptibility is further supported by our data, which are compatible with studies from [43] and Prabhurajeshwar and Kelmani [25]. Thus, it has been shown that the strain could be helpful as a probiotic or medication.

These nonhemolytic strains of *L. acidophilus* are commonly believed to be safer to eat and less prone to injuring tissue because our investigation revealed no hemolytic activity. Halder et al. [44] emphasized the safety and appropriateness of nonhemolytic probiotics for nutritional and medicinal uses, and our findings are consistent with their findings. According to oxgall testing, JB2CON is a good candidate for probiotic applications because of its high bile tolerance, essential for survival and function throughout the gastrointestinal system. These findings are identical to those of Ding and Shah [45], who found a similar reduction in probiotic strains following 8 h of bile salts treatment, and were confirmed by Ortakci et al. [23]. Identified JB2CON exhibits potential antibacterial activity against Gram‐negative bacteria, following Fijan′s [12] classification. Conversely, *C. albicans* did not demonstrate any measurable ZOI, which implies that the strain may have limited or no antifungal activity in the tested conditions. Our findings support Fijan [12], who found that *L. acidophilus* strains inhibited various infections to variable degrees. Nevertheless, the disc diffusion method results could only show a portion of the antibacterial activity of *L. acidophilus*. Despite being widely used for antimicrobial testing, the disc diffusion method provides narrower inhibition zones than other methods, such as the spot‐on grass agar method. Larger inhibition zones might result from improved antimicrobial activity expression made possible by the spot‐on grass agar technique′s ease of use and high sensitivity [46]. Thus, we hypothesize that this investigation may have seen a more substantial inhibitory effect, particularly against *E. coli* if the spot‐on grass agar technique had been used.

On TSA medium, we found TNTC for nonlactic contamination, but on SCA media at 35°C and 44°C, neither yeast nor mold growth was observed. The intricacy of microbial dynamics in probiotic dahi was highlighted by the appearance of yeast and mold colonies at room temperature. Although JB2CON may block some infections, mold and yeast contamination are always risky. In particular, *L. acidophilus* may act as a natural preservative by inhibiting the development of pathogenic microbes such as *Salmonella* sp., *P. aeruginosa*, *S. aureus*, and *E. coli* [47]. According to our research, the product may still be vulnerable to microbial contamination if appropriate measures are not taken, such as managing the environment and ensuring that fermentation and storage procedures are followed. To improve the safety and quality of probiotic dairy products, future studies should concentrate on comprehending nonlactic pollutants and creating plans to stop their proliferation [48]. The present study examined the effects of milk quality, namely protein content and milk age, on dahi′s titrable acidity and pH. We used cow milk with a fat content of at least 2% and a protein content of 3.5%, aligning with the ideal composition for full‐fat milk described by [49]. These properties provide a solid base for high‐quality dahi, with fat ensuring a creamy texture and protein enhancing the texture and consistency during fermentation [50].

This study tested probiotic dahi kept at refrigeration temperatures for JB2CON survival. The results exhibited that JB2CON has a significant bactericidal action against *E. coli* since no live *E. coli* cells were observed after 5.5 h. Based on the findings, Arepally et al. [51] revealed that *L. acidophilus*′s vitality was more significant at 4°C than at room temperature. However, *C. albicans* multiplied over 14 days, emphasizing the need to carefully select starter cultures and preparation processes to minimize fungal contamination. So, this study demonstrates the potential of probiotic dahi as a functional food with a 14‐day shelf life when cooked and kept correctly. In consumer acceptance of this dahi, all participants scored the texture neutrally, consistent with studies that texture relies on parameters such as raw milk content, incubation period, and additions. The texture of dahi depends on various factors, such as the composition of raw milk, incubation time, and additives [52]. These results imply that adjustments to the fermentation procedure and formulation can successfully produce a probiotic dahi with a mild texture and well‐balanced taste profile that is more palatable. This study demonstrates the critical probiotic potential of *L. acidophilus* (JB2CON), highlighting its advantageous qualities for immunological and gastrointestinal health. To establish *L. acidophilus* as a natural preservative in dairy products, the study also highlights its antibacterial action against Gram‐negative bacteria such as *E. coli*. The study also emphasizes how critical it is to have ideal fermentation and storage conditions to guard against microbial contamination and guarantee product safety.

## 5. Conclusion

The dahi in the current study was prepared from a potential probiotic, *L. acidophilus* JB2CON (OM909067), isolated from dahi. Given that the probiotic strain exhibited anti‐*E. coli* activity, this probiotic or probiotic dahi may provide an inexpensive option for recovering from and preventing diarrheal diseases and other *E. coli*‐related diseases in humans. This approach could save the costs and lives of billions globally who are infected with *E. coli*. This represents the most significant outcome of the research. Furthermore, our findings indicate that dahi contamination stemmed from unhygienic practices and unidentified starter cultures, which can reduce the shelf life of dahi and result in spoilage. Therefore, precautions should be taken, and dahi should be prepared with pasteurized milk. Considering contamination levels, probiotic count, pH, and taste, 14 days is an ideal shelf life for our probiotic dahi made from pasteurized milk.

## Author Contributions

Conceptualization: Sk. Md. Jakaria Al‐Mujahidy. Data curation: Sk. Md. Jakaria Al‐Mujahidy, Md. Liton Miah, and Shahidur Rahman. Formal analysis: Sk. Md. Jakaria Al‐Mujahidy, Md. Ramjan Sheikh, and Shahidur Rahman. Investigation: Md. Ekhlas Uddin. Methodology: Sk. Md. Jakaria Al‐Mujahidy. Writing original draft: Sk. Md. Jakaria Al‐Mujahidy and Md. Ramjan Sheikh. Writing—review and editing: Md. Ekhlas Uddin and Md. Ramjan Sheikh.

## Funding

The Dairy Development Research Project, Bangladesh Livestock Research Institute Regional Station, Baghabari, Shahjadpur, Sirajganj 6770, partially supported this work.

## Disclosure

This article is also visible as a reprint on an online website.

## Consent

The authors have nothing to report.

## Conflicts of Interest

The authors declare no conflicts of interest.

## Data Availability

Data will be provided upon request to the corresponding author.
